# Drought and Competition Mediate Mycorrhizal Colonization, Growth Rate, and Nutrient Uptake in Three *Artemisia* Species

**DOI:** 10.3390/microorganisms11010050

**Published:** 2022-12-23

**Authors:** David Eduardo Prado-Tarango, Ricardo Mata-Gonzalez, Matthew Hovland

**Affiliations:** Department of Animal and Rangeland Sciences, Oregon State University, Corvallis, OR 97331, USA

**Keywords:** *Artemisia*, *Taeniatherum caput-medusae*, arbuscular mycorrhizal fungi, stress amelioration, invasive species

## Abstract

The genus *Artemisia* includes several keystone shrub species that dominate the North American sagebrush steppe. Their growth, survival, and establishment are negatively affected by exotic invasive grasses such as *Taeniatherum caput-medusae*. While the outcomes of symbiotic relationships between *Artemisia* spp. and arbuscular mycorrhizal fungi (AMF) are ambiguous, the benefits of ameliorated nutrient and drought stress may be cryptic and better revealed under competition. We evaluated the effects of a commercial AMF inoculum on ameliorating biotic (competition with *T. caput-medusae*) and abiotic (drought) stress of *Artemisia tridentata* ssp. *wyomingensis*, *Artemisia arbuscula,* and *Artemisia nova* when grown in sterile and microbially active field soil. Stress amelioration was measured as an increase in biomass production and nutrient acquisition. Mycorrhizal colonization of roots was lower in *Artemisia* plants grown in competition, while *T. caput-medusae* colonization was higher in plants with greater moisture. Both types of stress negatively affected plant biomass. Commercial AMF inoculation did not increase biomass. Colonization from field soil increased average phosphorous concentration under drought for *A. tridentata* ssp. *wyomingensis* by 36% and *A. nova* by 125%. While commercial inoculum and live soil led to AMF colonization of *T. caput-medusae*, only the commercial inoculum increased average phosphorus uptake by 71%.

## 1. Introduction

Ecosystem deterioration is one of the most important problems in many areas around the world [1]. An approach to solve this problem is the development and implementation of ecological restoration programs [2,3]. In North America, one of the most threatened ecosystems is the sagebrush steppe where *Artemisia* species (a keystone plant species) have to endure competition with exotic annual grasses such as *Bromus tectorum* (cheatgrass), *Ventenata dubia* (ventenata), and *Taeniatherum caput-medusae* (medusahead) [4]. These annual species fiercely compete for water and nutrients with the perennial vegetation, especially at the seedling stage, quickly replacing the perennial cover with monocultures that eventually function as fuels for fire that further enhance their dominance [5,6]. Another challenge for plant species establishment is drought, which is a driver of successional processes in semi-arid systems and a barrier to the reestablishment of sagebrush species post-disturbance [7,8,9,10].

To survive in harsh environments, plant species have different resource acquisition strategies to be more or less competitive [11]. Yet, most competition studies are framed from an aboveground perspective, with limited focus on the soil community interactions, such as mycorrhizal fungi. Arbuscular mycorrhizal fungi (AMF) are a group of soil symbionts widely recognized for the mutualistic associations they develop within the roots of a vast number of terrestrial plant species [12,13]. Those associations might improve plant nutrient acquisition and regulate water uptake [12,14,15,16]. Because of this, AMF often mediate plant competition and are considered drivers of plant community structure [17]. However, AMF are often studied in crop systems with fast growing species [18,19,20], but questions remain about plant responsiveness to AMF in late-successional rangeland systems [21], where mycorrhizal associations may mediate ecosystem resistance to invasion of exotic annual plant species by enhancing native plant competitive abilities through stress amelioration [22].

Enhancing the competitive ability of perennial native shrubs using AMF during the establishment phase could be a key step for restoration programs [23]. However, the response of invasive exotic grasses to AMF also requires further research because *B. tectorum* and *T. caput-medusae* have shown ambiguous results to AMF [24,25]. Furthermore, the long-term presence of invasive species with low or no mycorrhizal dependency might result in modification of the soil microbial communities [26,27]. Thus, in scenarios where local AMF might be absent or degraded, introduction of AMF from an external inoculum source might be required to allow native plant establishment, but it is also important to understand the response of invasive plant species such as *T. caput-medusae* since inoculation could potentially affect the dynamics of competition. 

Important native plant species from sagebrush steppe include *Artemisia tridentata* ssp. *wyomingensis* Beetle and Young (Wyoming big sagebrush), *Artemisia arbuscula* Nutt (low sagebrush), and *Artemisia nova* A. Nelson (black sagebrush). *A. tridentata* ssp. *wyomingensis* is a known mycorrhizal plant that has been extensively studied for restoration, although mycorrhizal responsiveness is ambiguous, while *A. arbuscula* and *A. nova* have been much less studied [28,29,30,31]. All of those species are important components of sagebrush steppe ecosystems and understanding their responses would inform restoration practices. 

We evaluated the effect of a commercial AMF inoculum on the competitive ability of three sagebrush shrubs (*A. tridentata* ssp. *wyomingensis*, *A. arbuscula*, and *A. nova*) during the establishment phase while facing biotic (intraspecific competition from *T. caput-medusae*) and abiotic (drought) stress. We hypothesized that: (1) a commercial AMF inoculum ameliorates stress in *Artemisia* spp. imposed by competition from *T. caput-medusae* and drought, and (2) *T. caput-medusae* is not more competitive under mycorrhizal conditions.

## 2. Materials and Methods

A competition experiment with *A. tridentata* ssp. *wyomingensis*, *A. arbuscula*, *A. nova,* and *T. caput-medusae* was conducted in a greenhouse at Oregon State University. This experiment was a 3 × 2 × 2 × 2 × 2 factorial pot design with five replicates in a complete randomized block arrangement. The factors were plant species (*A. tridentata* ssp. *wyomingensis*, *A. arbuscula*, *A. nova*), soil type (live field soil or autoclaved field soil), moisture levels (40% and 80% field capacity), competition (presence/absence of *T. caput-medusae*), and inoculum type (commercial AMF or a “sham” inoculum). Sham inoculum is the autoclaved substrate (field soil or AMF inoculum) in which we reincorporated a microbial wash to have a media with the soil organisms except for AMF and was included as a control to compare the mycorrhizal effects only [32]. For the complete list of treatment combinations, see Table 1. To account for temperature and light gradients in the greenhouse, we used each replicate as a block.

Seeds of *A. tridentata* ssp. *wyomingensis*, *A. nova,* and *A. arbuscula* were obtained from commercial vendors during the summer of 2017, and seeds of *T. caput-medusae* were collected from the Crooked River National Grasslands during the fall of 2018. Field soil was obtained from the same area where *T. caput-medusae* was collected, a disturbed sagebrush grassland ecosystem. Coordinates where the soil was sampled are 44°27′50″ N–121°0′57″ W. The collected soil had a loam texture (44% sand, 22% clay and 34% silt) and a Munsell color 10YR 4/3 (dry) and 10YR 2/2 (wet). The mycorrhizal inoculum was AM120 standard (Reforestation Technologies International, Gilroy, CA, USA). Label contents of inoculum are 100% *Rhizophagus intraradices*. 

Containers were plastic Poly-tainer™ cans of 2.41 L (0.65 gal), 15.2 cm (6 in) diameter and 17 cm (7 in) depth. Containers were steam sterilized at 80 °C for 2 h to remove possible contaminants. We added 2 kg of soil to each pot (autoclaved or field), then a layer of 100 g of either mycorrhizal inoculum or sham inoculum, followed by another layer of 100 g of soil. Seeds were sown and kept under constant moisture for two weeks to ensure germination. Multiple seeds were sown in each pot for *A. tridentata* ssp. *wyomingensis*, *A. arbuscula* and *A. nova*. After germination, only one seedling was left per pot. Moisture treatments began after the 2-week period. Soil moisture treatments (40% and 80% field capacity) were determined using the gravimetric method [33] based on the % of soil moisture of field capacity. Percentage of soil water content was determined by manually weighting the pots.

Competition was simulated by growing each of the *Artemisia* species with 15 individuals of *T. caput medusae* for a density of 600 plants m^−2^ [34]. Twenty seeds of *T. caput-medusae* were sown in each pot, and only fifteen were left. For each combination, we had 5 replicates, giving a total of 240 pots in the study. 

To account for the microbial communities from the field soil and possibly from the inoculum substrate, we made a microbial wash that was added to sterilized soil to create a sham inoculum with all microbial communities except for AMF from both soil and inoculum [35]. Microbial wash was made by using 700 g of the substrate (field soil or inoculum). We made a slurry by adding the soil to 4 L of water, stirred, and allowed to settle for 30 min. The settled solution was poured through a 45 μm sieve, then again through a 25 μm sieve three times, settled for other 30 min, and finally was poured through a 20 μm sieve [36]. The final solution was brought to a final volume of 13 L and 100 mL, and 10 mL of microbial wash were added to all treatments composed of autoclaved soil. Sham inoculum was made by autoclaving mycorrhizal inoculum and adding 10 mL of the microbial wash to the sterilized inoculum to produce a media with all the inoculum components except for mycorrhizal spores [35]. 

All plants were grown for 6 months (26 weeks). Moisture treatments began after 2 weeks of emergence of germinated seeds to allow establishment of at least one individual in each pot. After the growing period, all plants were manually harvested. Shoots were cut and stored in paper bags. Roots were carefully extracted from the soil, washed, and a subsample was stored in 20 mL vials containing a mixture of 50% ethanol and 5% acetic acid for root colonization measurements. 

Roots for colonization were cleared and stained: roots were incubated at 90 °C in 5% (*w*/*v*) KOH for 20 min, then rinsed with warm water, followed by an incubation period of 10 min in 1% HCL, incubated then in trypan blue stain (0.5% *w*/*v* trypan blue, 5% *v*/*v* lactic acid, 50% *v*/*v* glycerol) for 20 min, rinsed with warm water, and incubated in de-stain (5% lactic acid, 50% glycerol) overnight at room temperature [37]. The stained roots were mounted onto microscope slides to assess for mycorrhizal colonization [37,38]. Roots were considered colonized if they contained hyphae, vesicles, or arbuscules within the root cortex.

We measured plant biomass production (shoot dry weight, root dry weight, total dry biomass), root colonization, and plant nutrient content (N, P, K). Plant nutrient analysis was conducted at the Soil Health Laboratory at Oregon State University. N was analyzed by dry combustion with a direct measure of total nitrogen by Elemental Macro Cube analyzer (vario macro cube, elementar, Germany). Soil nutrients P and K were extracted using a Mehlich 3 solution and quantified on Agilent 5110 ICP-OES. Mycorrhizal effects were subsampled from a fraction of the root system, the rest was used to estimate dry weight. Because of the small root size for all *Artemisia* species under competition, colonization data were evaluated using the entire root system and root weight was reported only as fresh root weight. 

Data was analyzed using R (2021) with a separate factorial ANOVA for each plant species using soil type (autoclaved or field soil), moisture level (high or low), inoculum (sham or commercial inoculum), and competition (presence/absence of *T. caput-medusae*) as main effects and block as a random effect. Before analysis, variables that did not meet the normality and equal variance were log10 (x + 1) transformed in order to fulfill assumptions of the model. 

## 3. Results

### 3.1. AMF Root Colonization of Artemisia Species

**Figure 1:**
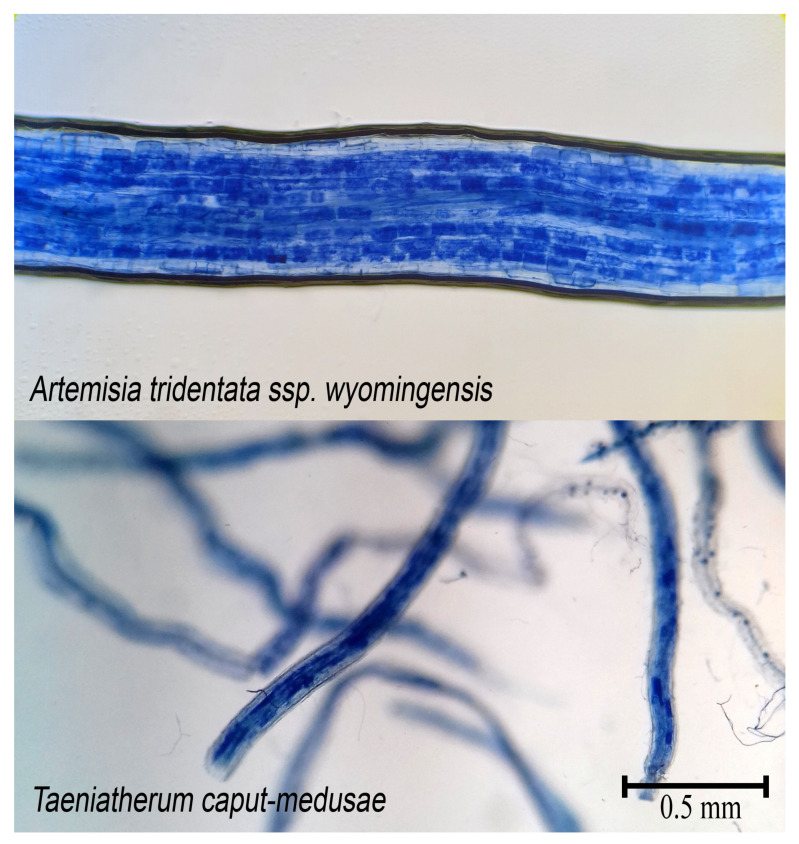
Cleared and stained roots of *Artemisia tridentata* ssp. *wyomingensis* and *Taeniatherum caput-medusae*. Arbuscules (dark blue) and hyphae from AMF can be seen colonizing cortical cells.

AMF colonization for all three *Artemisia* species varied by soil type (*p* < 0.001, F = 85.39), AMF inoculation (*p* < 0.001, F = 26.56), and presence/absence of competition (*p* < 0.001, F = 94.75) but not by moisture level (*p* = 0.21, F = 1.53). In general, drought and competition resulted in lower colonization, and inoculum addition resulted in a significant colonization increase in autoclaved soils. Colonization was present in inoculated plants (Figure 1) and was zero in all plants grown in autoclaved soils with sham inoculum.

The species *A. tridentata* ssp. *wyomingensis* was colonized in both field and inoculated field soils under both moisture levels, and also from inoculated autoclaved soils with high moisture only (Table 2). However, inoculation did not significantly increase average colonization in field soils with high moisture levels regardless of competition (*p* > 0.05). Inoculated plants grown in field soils with both competition and drought had a significant increase in colonization compared to sham-inoculated plants from field soils with competition and drought stress (*p* = 0.002). Still, this level of colonization was significantly less compared to sham-inoculated plants grown in field soils, under drought, and without competition (*p* < 0.001). For *A. arbuscula*, the combination of drought and competition in inoculated plants grown in field soils resulted in a significant decrease in colonization (*p* = 0.04) (Table 2). Interestingly, drought and competition resulted in a positive significant increase in colonization in inoculated plants grown in autoclaved soil (*p* = 0.007). The species *A. nova* did not respond with colonization increases with the addition of the inoculum (*p* > 0.05). The presence of colonization was higher in field soils (Table 2). However, we found a significant colonization increase when autoclaved soils were inoculated and subjected to drought with no competition compared to uninoculated autoclaved treatments (*p* < 0.001). The addition of competition to autoclaved inoculated soils with drought resulted in zero % of colonization for *A. tridentata* ssp. *wyomingensis* and *A. nova*. 

### 3.2. AMF Root Colonization of the Invasive T. caput medusae

Colonization of *T. caput-medusae* did not statistically differ when grown as competition with different *Artemisia* species (*p* = 0.55, F = 0.58). Colonization of this species was driven by differences in soil (*p* < 0.001, F = 193.05), moisture (*p* = 0.01, F = 3.75), and inoculum application (*p* < 0.001, F = 55.75). Colonization was greater in high moisture treatments compared to low moisture (Table 3). Inoculation of autoclaved soils in both moisture levels resulted in an increase of colonization compared to sham-inoculated treatments (*p* < 0.05). When *T. caput-medusae* was grown with *A. tridentata* ssp. *wyomingensis* and *A. arbuscula*, inoculation of field soils did not result in significant changes of colonization. However, colonization was greater in low moisture treatments when grown in inoculated field soils with *A. nova.*

### 3.3. Biomass Production of the 3 Artemisia Species

**Figure 2:**
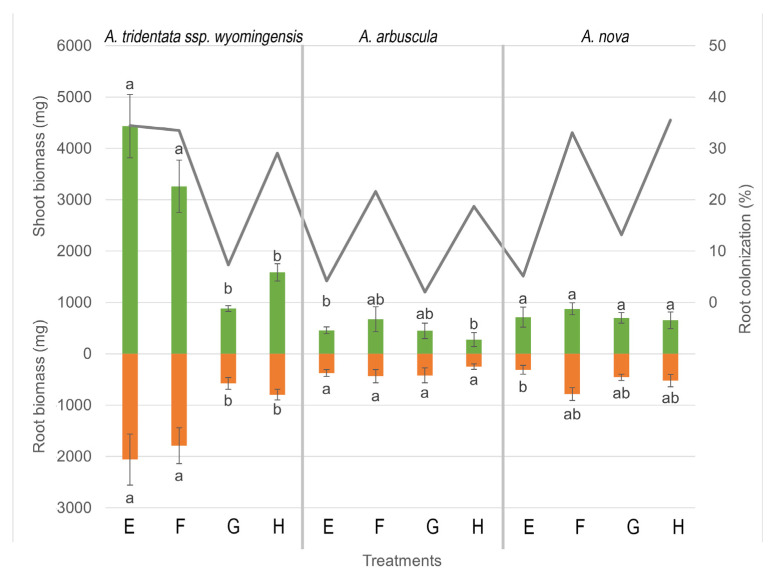
Shoot and root biomass (bars) and root colonization (lines) ±SE (error bars) of 3 species of *Artemisia* grown under different combinations of treatments (soil, moisture, and AMF inoculation) without competition for 21 weeks. Different letters above and below bars indicate significant differences among treatments (Tukey’s HSD). All treatments have commercial AMF inoculum, sham treatments not shown. Treatments: (E) high moisture, autoclaved soil; (F) high moisture, field soil; (G) low moisture, autoclaved soil; (H) low moisture, field soil.

**Figure 3:**
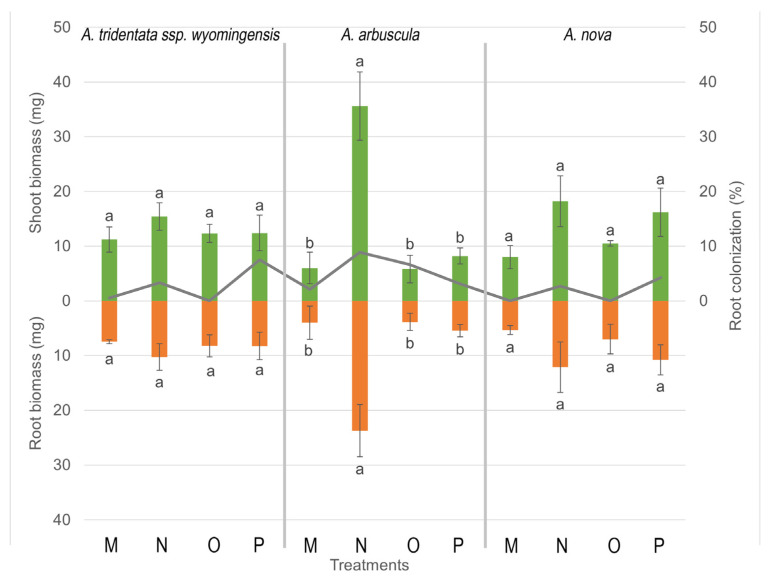
Shoot and root biomass (bars) and root colonization (lines) ±SE (error bars) of 3 species of Artemisia grown under different combinations of treatments (soil, moisture, and AMF inoculation) growing in competition with *Taeniatherum caput-medusae* for 21 weeks. Different letters above and below bars indicate significant differences among treatments (Tukey’s HSD). All treatments have commercial AMF inoculum, sham treatments not shown. Treatments: (M) high moisture, autoclaved soil; (N) high moisture, field soil; (O) low moisture, autoclaved soil; (P) low moisture, field soil.

Competition was the main driver of shoot, root, and total biomass production for *A. tridentata* ssp. *wyomingensis* (*p* < 0.001, F = 90.11, 71.01 and 826.65), *A. arbuscula* (*p* < 0.001, F = 63.54, 40.24 and 42.81), and *A. nova* (*p* < 0.001, F = 133.68, 89 and 6.49). All 3 *Artemisia* species produced more biomass when grown alone, compared to plants grown with competition (Figure 2 and Figure 3). Moisture was also associated with the production of total biomass for *A. tridentata* ssp. *wyomingensis* (*p* < 0.001, F = 26.94), *A. arbuscula* (*p* = 0.01, F = 6.18), and *A. nova* (*p* = 0.01, F = 6.49) and with shoot/root production for *A. tridentata* ssp. *wyomingensis* (*p* < 0.001, F = 24.72 and 21.21) and *A. arbuscula* (*p* = 0.003 and 0.03, F = 9.41 and 4.60). Drought treatments resulted in a decrease of biomass production in all three species (Figure 2). Furthermore, the combination of drought and competition resulted in the lowest production of biomass for all three shrub species (Figure 3). At the same time, inoculation was not found to be significant in increasing shoot, root, or total biomass production of any species (*p* > 0.05), when compared to non-inoculated treatments. Inoculation decreased shoot growth of *A. arbuscula* and root growth of *A. nova* when grown without competition in high moisture levels (Figure 2). Soil type was only significant for total and root biomass of *A. nova* (*p* = 0.007 and 0.05, F = 7.50 and 3.93) as field soils produced more biomass without competition. From the three species, *A. arbuscula* produced the greatest shoot and root biomass when grown in competition at high moisture levels regardless of inoculation (Figure 3).

ANOVA revealed that colonization was related to total and shoot biomass production for *A*. *tridentata* ssp. *wyomingensis* (*p* < 0.001, F = 37.83 and 36.66) and *A. nova* (*p* < 0.001, F = 28.25 and 38.93) and to root biomass for *A*. *tridentata* ssp. *wyomingensis* (*p* < 0.001, F = 29.84). With the exception of sham-inoculated autoclaved soil, treatments with higher shoot and total biomass production were also the treatments with highest colonization for both *A*. *tridentata* ssp. *wyomingensis* and *A. nova*. 

### 3.4. Biomass Production of T. caput medusae

**Figure 4:**
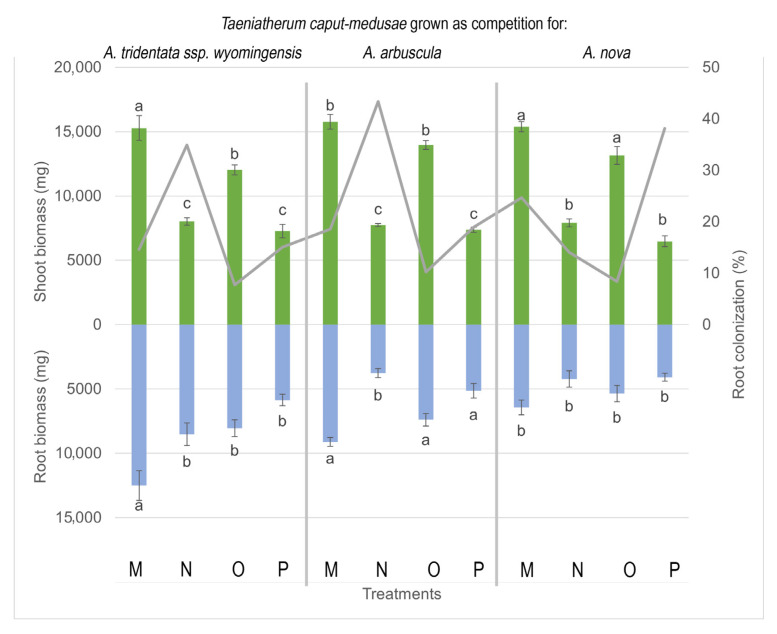
Shoot and root biomass (bars) and root colonization (lines) ±SE (error bars) of *Taeniatherum caput-medusae* grown in competition with 3 species of *Artemisia* under different combinations of treatments (soil, moisture, and AMF inoculation) for 21 weeks. Different letters above and below bars indicate significant differences among treatments (Tukey’s HSD). All treatments have commercial AMF inoculum, sham treatments not shown. Treatments: (M) high moisture, autoclaved soil; (N) high moisture, field soil; (O) low moisture, autoclaved soil; (P) low moisture, field soil.

*T. caput-medusae* total biomass responded to the different *Artemisia* species (*p* < 0.001, F = 9.35), soil type (*p* < 0.001, F = 330.72), moisture level (*p* < 0.001, F = 24.14), and inoculum (*p* = 0.01, F = 5.96). Shoot biomass responded to soil type (*p* < 0.001, F = 44.59), moisture level (*p* < 0.001, F = 745.91), and inoculum type (*p* = 0.03, F = 4.59). Root production varied by associated *Artemisia* species (*p* < 0.001, F = 26.13), soil type (*p* < 0.001, F = 84.28), moisture (*p* = 0.004, F = 8.37), and inoculum type (*p* = 0.03, F = 4.53). Total, shoot, and root biomass production were greater in *T. caput medusae* grown in autoclaved soils, while field soils always had lower biomass production (Figure 4). Drought treatments reduced shoot biomass production in *T. caput-medusae* when associated with *A. tridentata* ssp. *wyomingensis* and *A. arbuscula*. Finally, inoculation resulted in lower shoot biomass production in *T. caput-medusae* when grown in association with *A. nova* in field soils and under drought.

### 3.5. Nutrient Acquisition of the 3 Artemisia Species

**Figure 5:**
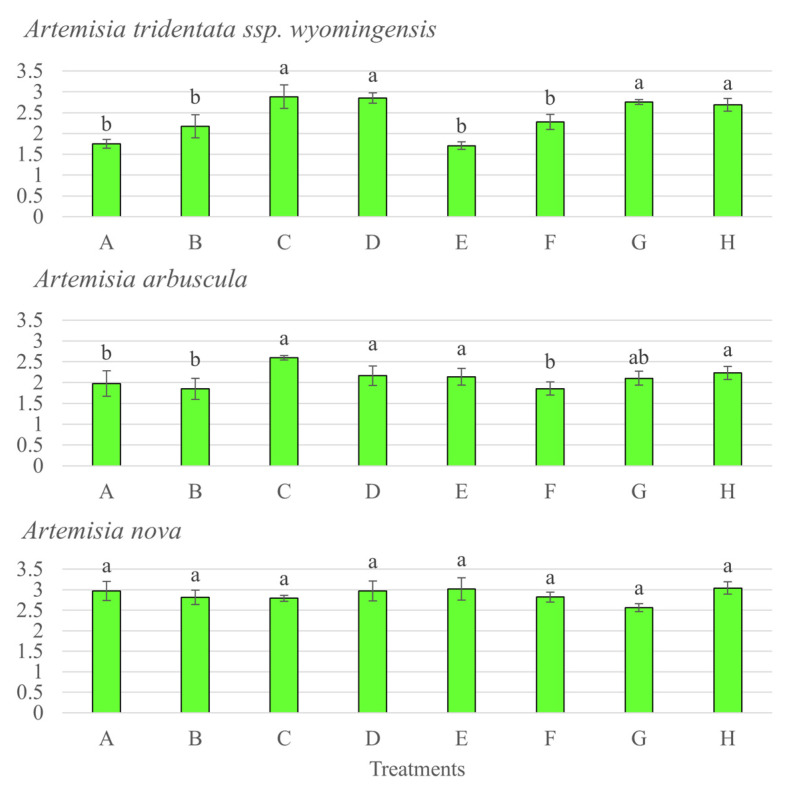
Nitrogen (N) concentration ±SE (error bars) in *Artemisia tridentata* ssp. *wyomingensis*, *Artemisia arbuscula,* and *Artemisia nova*. Different letters above bars indicate significant differences among treatments (Tukey’s HSD). N content of *Artemisia* species grown under competition not measured.

**Figure 6:**
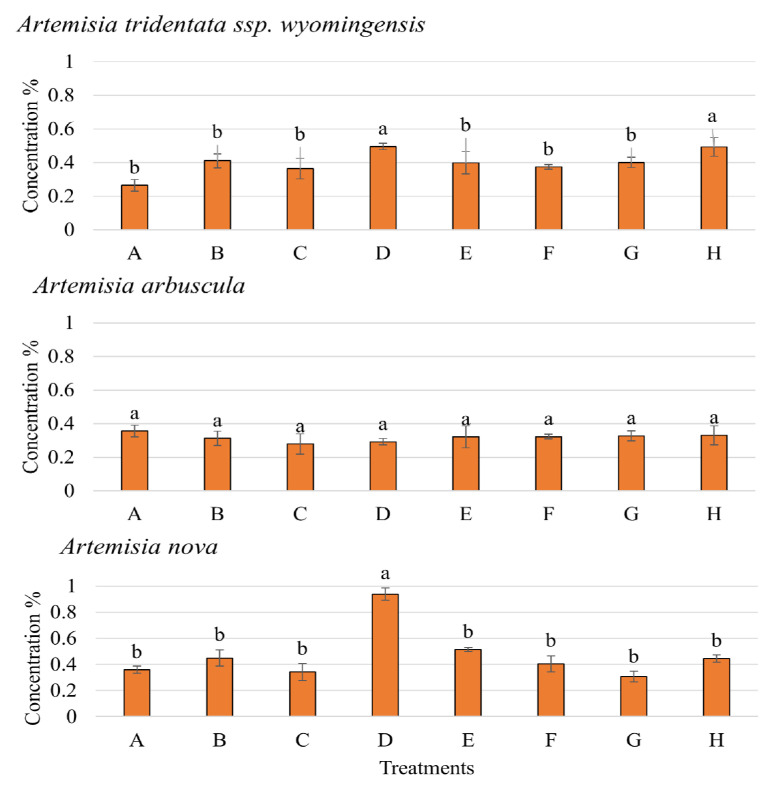
Phosphorus (P) concentration ±SE (error bars) in *Artemisia tridentata* ssp. *wyomingensis*, *Artemisia arbuscula,* and *Artemisia nova* and their associated *Taeniatherum caput-medusae* grown as competition. Different letters above bars indicate significant differences among treatments (Tukey’s HSD). P content of *Artemisia* species grown under competition not measured.

**Figure 7:**
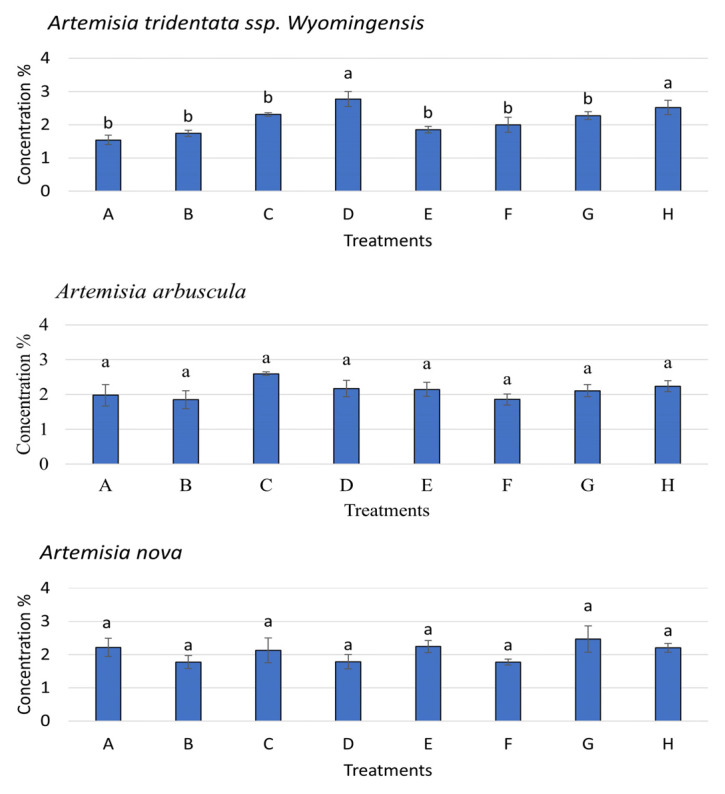
Potassium (K) concentration ±SE (error bars) in *Artemisia tridentata* ssp. *wyomingensis*, *Artemisia arbuscula*, and *Artemisia nova*. Different letters above bars indicate significant differences among treatments (Tukey’s HSD). K content of *Artemisia* species grown under competition not measured.

Nutrient acquisition for the *Artemisia* species was measured without competition only since plants grown under competition did not produce enough biomass for nutrient analysis. N content of *A. tridentata* ssp. *wyomingensis* significantly varied between treatment combinations (*p* < 0.001, F = 7.258), with moisture level being more significant (*p* < 0.001, F = 39.48) than soil type (*p* = 0.10, F = 2.89) and inoculum (*p* = 0.66, F = 0.196). N content was greater in low moisture treatments, regardless of soil or inoculum type (Figure 5). *A. arbuscula* N content responded significantly to the interaction of moisture level and soil type (*p* < 0.001, F = 39.65). N content of *A. nova* did not significantly change among any soil, moisture, or inoculation treatments (*p* > 0.05). P content in *A. tridentata* ssp. *wyomingensis* significantly varied by moisture level (*p* = 0.03, F = 5.41) and soil type (*p* = 0.01, F = 6.86). P content was greater when grown at low moisture in field soils with both sham and AMF inoculums (0.49% and 0.49%) compared to all other treatments (Figure 6). *A. arbuscula* had no significant differences in P content for any treatments or interactions (*p* > 0.05). *A. nova* had a greater P content when grown with low moisture and field soils with sham inoculum (*p* < 0.05, F = 5.41). K content in *A. tridentata* ssp. *wyomingensis* statistically responded to moisture level only (*p* < 0.001, F = 39.31), as it was greater when grown in low moisture, field soils with sham and AMF inoculums (2.77% and 2.52%) (Figure 7). K content in *A. arbuscula* and *A. nova* had no significant differences among treatments (*p* > 0.05). 

### 3.6. Nutrient Acquisition of T. caput-medusae

Nutrient content measured in *T. caput-medusae* grown as competition for the three *Artemisia* species is shown in Table 4. N content of *T. caput-medusae* when grown with *A. tridentata* ssp. *wyomingensis* responded to the interaction of moisture level and inoculum (*p* < 0.001, F = 27.57), as it was greater when grown at low moisture, in autoclaved soil, and with commercial inoculum (0.70%). N content of *T. caput-medusae* associated with *A. arbuscula* responded to a greater extent to the interaction of moisture and soil (*p* < 0.001, F = 39.55) as it was greater in field soils with high moisture. When grown with *A. nova*, N concentration responded to inoculum (*p* < 0.001, F = 10.35) and the interaction of soil and moisture (*p* < 0.001, F = 25.04) as it was greater when inoculated with commercial AMF, at high moisture, and in both soil types (0.82% and 0.70).

P concentration of *T. caput-medusae* grown with *A. tridentata* ssp. *wyomingensis* significantly varied by moisture level (*p* = 0.004, F = 10.95) and the interaction of moisture and soil (*p* < 0.001, F = 40.63). P content was greater at high moisture levels in field soil with sham and AMF inoculums (0.12% and 0.10%) and in low moisture, autoclaved soils with AMF inoculum (Table 4). P concentration when grown with *A. arbuscula* varied by moisture level (*p* = 0.01, F = 8.15), soil type (*p* = 0.03, F = 5.46), and the interaction of moisture and soil (*p* < 0.001, F = 34.75), as P concentration increased when grown in both high and low moisture, field and autoclaved soils, and inoculated with commercial AMF. Finally, when grown associated with *A. nova*, P content of *T. caput-medusae* reported no significant differences in any treatments (*p* > 0.05). 

K content in *T. caput-medusae* grown with *A. tridentata* and *A. nova* varied by moisture (*p* = 0.01 and 0.002, F = 6.86 and 12.15) and soil type (*p* < 0.01 and 0.002, F = 75.35 and 12.27). K was greater in high moisture, autoclaved soils, and inoculum (0.59% and 0.73%) (Table 4). K content when associated to *A. arbuscula* responded to moisture (*p* < 0.001, F = 16.51), soil type (*p* < 0.001, F = 24.61), inoculum (*p* = 0.02, F = 5.79), and the interaction of moisture and soil (*p* < 0.001, F = 8.74).

## 4. Discussion

### 4.1. Can Environmental Stress Be Ameliorated with a Commercial AMF Inoculum?

We tested the hypothesis that by using a commercial AMF inoculum we would ameliorate biotic and abiotic stress in three *Artemisia* species, and that such inoculum would not make *T. caput-medusae* more competitive. Based on our results, we cannot support either hypothesis as we did not find evidence that by using a commercial AMF inoculum we can effectively ameliorate biotic (intraspecific competition) or abiotic (drought) stress in the tested *Artemisia* species. We also found evidence of nutrient increases for *T. caput-medusae* with the addition of the commercial inoculum. 

Biomass production of the *Artemisia* species was affected by competition and drought rather than AMF inoculation. Drought is a widely recognized factor that affects plant growth [10,39]. Plants subjected to drought typically reflect a lower production of biomass as the water reduction from soil to the plant decreases cell division and eventually is reflected in less biomass production, including the roots [8]. Some plant species allocate more biomass to the roots trying to acquire resources as a survival strategy, but when the plant species are subject to both drought and competition, this strategy might not be sufficient [40]. Competition (interspecific or intraspecific) negatively affects the plants species growth by reducing the shoot’s ability to photosynthesize or lessening the nutrient availability in the soil by neighboring plants [11]. A high-root-density environment can create nutrient depletion zones in the soil, reducing the benefits from the AMF symbiosis and favoring the plant species with faster nutrient acquisition strategies [11,40]. Therefore, the AMF benefits are diminished in a high competition scenario and the nutrient acquisition strategies became more important for growth. In this context, all three *Artemisia* species were outcompeted by the faster shoot and root growth from *T. caput-medusae*, which reduced moisture access to the roots and likely created a nutrient depleted environment for the *Artemisia* seedlings to grow even though they were colonized by AMF. 

Furthermore, the mycorrhizal symbiosis represents a considerable carbon cost for the host, which might be too much for a newly emerged seedling under a competitive environment [12,41]. When the AMF colonizes the plant and it is unable to provide additional nutrients, the relationship between host and fungi becomes parasitic [42]. This response is also dependent on the AMF species or strains composition off the inoculum or even pathogens in the soil [23,43]. Additional evidence that competition was a major driver for the observed plant responses is that *Artemisia tridentata* ssp. *wyomingensis*, and *A. nova* received N, P, and K gains from AMF colonization from live field soils subjected to drought without competition. This corresponds with the stress-gradient hypothesis, suggesting that mutualism is promoted under certain levels of stress [44]. In this scenario, AMF provides nutrient benefits when environmental stress such as drought reduces the plants ability to access nutrients [12,15,16,45]. However, when the *Artemisia* plants were subjected to both types of stress, the AMF symbiosis did not provide any measurable effect. However, further research is required to validate this hypothesis.

These results are in contrast to some studies suggesting that mycorrhizal inoculation provided growth benefits [46] as well as nutrient increases [45,47]. Multiple studies reporting positive mycorrhizal benefits evaluated fast-growing plants, members of the Fabaceae family [19,39], or crop species [18], while other studies have used AMF inoculum composed of multiple species or cultivated from undisturbed ecosystems [28,31]. This differs from our study in which our commercial inoculum was reportedly composed of only *Rhizophagus intraradices,* and the field soil was obtained from a disturbed site. Furthermore, other studies suggest that each AMF inoculum type has a different response even in the same plant species [29]. For slow-growing, late seral species such as *A. tridentata* ssp. *wyomingensis*, *A. arbuscula,* and *A. nova* direct mycorrhizal benefits might depend on differential AMF communities that were not present in the inoculum or in the soil.

### 4.2. Mycorrhizal Responsiveness of T. caput-medusae

Our findings suggest that *T. caput-medusae* responds as a facultative mycorrhizal plant. *T. caput medusae* biomass was greater in soils without AMF, and the re-addition of the soil microbial community provided no measurable effect. Other studies also ruled out the effect of the soil microbial and bacterial communities as possible drivers of *T. caput-medusae* invasiveness [48]. Previous studies with *T. caput-medusae* found similar results when colonized from field soils, having a lower biomass production compared to autoclaved soils [21]. We expected our species to be colonized in a greater extent when inoculated with a commercial AMF inoculum; instead, all species presented higher colonization in live field soils including *T. caput-medusae*. However, why might a facultative mycorrhizal plant exhibit greater AMF colonization than an obligate AMF species? Facultative mycorrhizal species are able to associate with several AMF species and maintain low levels of colonization which might increase during stress periods to obtain benefits and become established even in degraded soils [49]. Some invasive species such as cheatgrass (*Bromus tectorum*) are known for their response to stressors such as drought and competition by allocating less resources to biomass and more to reproduction [29]. So, even in high competition environments, *T. caput-medusae* can effectively compete and become established. Further studies are required to understand this response such as a continuous evaluation of *T. caput-medusae* on the field AMF densities over time. Finally, we cannot discard the possibility of a particular species or strain of AMF present in our field site that could be driving such growth depression in *T. caput-medusae* and further studies on fungal communities are required to test such hypotheses.

## 5. Conclusions

We were unable to accept our original hypotheses that a commercial AMF inoculum would reduce stress on the *Artemisia* species, but we found evidence of mycorrhizal benefits from live field soils in three *Artemisia* species without competition and from commercial AMF inoculum on *T. caput-medusae*. Furthermore, stressful conditions such as competition and drought mediated AMF colonization and growth responses on the *Artemisia* plant species. The effect of AMF promoting plant growth with and without stress has been previously studied mostly on crops species but not so much on rangeland plant species. Even though plant biomass did not reflect direct benefits (biomass increases) associated with nutrient acquisition in stressful conditions, a constant increase in non-mobile nutrients may be associated to long term plant establishment or fertility, which eventually might result in a more competitive plant. Proper maintenance of P supply has an important role in plant hydraulic connectivity and water uptake and hence resistance to stress.

Inoculation using external AMF sources has been a debated topic as their effects on invasive species are not completely clear. Our research provides valuable information for restoration in rangelands that face invasion with invasive species such as *T. caput-medusae*. We found evidence that *T. caput-medusae* received N, P, and K increases when inoculated with the commercial AMF. This finding implies that AMF inoculation (at least an inoculum composed of *Rhizophagus intraradices*) might not be recommended in rangelands in which *T. caput-medusae* is present. However, this response needs to be further evaluated, particularly under field conditions.

## Figures and Tables

**Table 1:** Different combinations of 16 treatments (high-low moisture, autoclaved-field soils, sham-AMF inoculum, no-yes competition) used in the study.

Letter	Treatment Combinations
A	High moisture, autoclaved soil, sham inoculum, no competition
B	High moisture, field soil, sham inoculum, no competition
C	Low moisture, autoclaved soil, sham inoculum, no competition
D	Low moisture, field soil, sham inoculum, no competition
E	High moisture, autoclaved soil, commercial AMF inoculum, no competition
F	High moisture, field soil, commercial AMF inoculum, no competition
G	Low moisture, autoclaved soil, commercial AMF inoculum, no competition
H	Low moisture, field soil, commercial AMF inoculum, no competition
I	High moisture, autoclaved soil, sham inoculum, competition
J	High moisture, field soil, sham inoculum, competition
K	Low moisture, autoclaved soil, sham inoculum, competition
L	Low moisture, field soil, sham inoculum, competition
M	High moisture, autoclaved soil, commercial AMF inoculum, competition
N	High moisture, field soil, commercial AMF inoculum, competition
O	Low moisture, autoclaved soil, commercial AMF inoculum, competition
P	Low moisture, field soil, commercial AMF inoculum, competition

**Table 2:** Mean percentages and SE of AMF colonization in roots of *Artemisia tridentata* ssp. *wyomingensis*, *A. arbuscula,* and *A. nova* grown for 6 months in different soil, moisture, inoculum, and competition treatments.

Treatments				Colonization ± SE		
Moisture	Soil	Inoculum	Competition	*A. tridentata*	*A. arbuscula*	*A. nova*
High	Autoclaved	Sham	No	0.00 ± 0.00	0.00 ± 0.00	0.00 ± 0.00
High	Field	Sham	No	37.85 ± 4.71	8.19 ± 2.72	33.8 ± 4.91
Low	Autoclaved	Sham	No	0.00 ± 0.00	0.00 ± 0.00	0.00 ± 0.00
Low	Field	Sham	No	28.59 ± 4.06	17.61 ± 2.14	24.7 ± 1.53
High	Autoclaved	AMF	No	34.42 ± 7.02	4.19 ± 3.03	5.14 ± 5.14
High	Field	AMF	No	33.50 ± 2.52	221.61 ± 1.3	33 ± 2.42
Low	Autoclaved	AMF	No	7.32 ± 2.71	2.03 ± 2.03	13.2 ± 0.63
Low	Field	AMF	No	29.10 ± 2.27	18.73 ± 6.00	35.5 ± 2.64
High	Autoclaved	Sham	Yes	0.00 ± 0.00	0.00 ± 0.00	0.00 ± 0.00
High	Field	Sham	Yes	2.04 ± 0.97	9.59 ± 2.60	2.75 ± 1.66
Low	Autoclaved	Sham	Yes	0.00 ± 0.00	0.00 ± 0.00	0.00 ± 0.00
Low	Field	Sham	Yes	1.59 ± 0.77	1.01 ± 0.71	2.11 ± 1.46
High	Autoclaved	AMF	Yes	0.54 ± 0.37	2.10 ± 2.10	0.00 ± 0.00
High	Field	AMF	Yes	3.30 ± 1.19	8.89 ± 1.87	2.65 ± 1.33
Low	Autoclaved	AMF	Yes	0.00 ± 0.00	6.63 ± 1.20	0.00 ± 0.00
Low	Field	AMF	Yes	7.52 ± 2.31	3.10 ± 0.51	4.23 ± 1.31

**Table 3:** Mean percentages and SE of AMF colonization in roots of *Taeniatherum caput-medusae* grown for 6 months in different soil, moisture, inoculum treatments, and associated *Artemisia* species.

*T. caput-medusae* grown with *A. tridentata* ssp. *wyomingensis*		
Treatment	Colonization %	SE
High moisture, autoclaved soil, sham inoculum, competition	0.00	0.00
High moisture, field soil, sham inoculum, competition	28.66	2.11
Low moisture, autoclaved soil, sham inoculum, competition	0.00	0.00
Low moisture, field soil, sham inoculum, competition	27.18	0.57
High moisture, autoclaved soil, AMF inoculum, competition	14.60	2.39
High moisture, field soil, AMF inoculum, competition	34.88	1.89
Low moisture, autoclaved soil, AMF inoculum, competition	7.74	2.10
Low moisture, field soil, AMF inoculum, competition	15.04	2.28
*T. caput-medusae* grown with *A. arbuscula*		
Treatment	Colonization %	SE
High moisture, autoclaved soil, sham inoculum, competition	0.00	0.00
High moisture, field soil, sham inoculum, competition	61.40	4.65
Low moisture, autoclaved soil, sham inoculum, competition	0.00	0.00
Low moisture, field soil, sham inoculum, competition	25.59	1.74
High moisture, autoclaved soil, AMF inoculum, competition	18.58	2.16
High moisture, field soil, AMF inoculum, competition	43.35	2.40
Low moisture, autoclaved soil, AMF inoculum, competition	10.24	3.55
Low moisture, field soil, AMF inoculum, competition	18.92	2.10
*T. caput-medusae* grown with *A. nova*		
Treatment	Colonization %	SE
High moisture, autoclaved soil, sham inoculum, competition	0.00	0.00
High moisture, field soil, sham inoculum, competition	43.82	1.53
Low moisture, autoclaved soil, sham inoculum, competition	0.00	0.00
Low moisture, field soil, sham inoculum, competition	23.13	2.07
High moisture, autoclaved soil, AMF inoculum, competition	24.68	1.38
High moisture, field soil, AMF inoculum, competition	14.06	2.55
Low moisture, autoclaved soil, AMF inoculum, competition	8.38	2.24
Low moisture, field soil, AMF inoculum, competition	38.14	1.50

**Table 4:** Mean N, P, and K concentration ±SE in *Taeniatherum caput-medusae* grown for 6 months in different soil, moisture, inoculum treatments, and *Artemisia* species. Different letters in each column indicate significant differences among treatments (Tukey’s HSD).

*A. tridentata*	Treatment	N (%)	SE	P (%)	SE	K (%)	SE
	I	0.54 ^b^	0.01	0.74 ^ab^	0.01	0.39 ^b^	0.06
	J	0.68 ^b^	0.02	0.12 ^a^	0.006	0.39 ^b^	0.03
	K	0.68 ^b^	0.04	0.09 ^ab^	0.01	0.43 ^b^	0.05
	L	0.55 ^b^	0.01	0.07 ^ab^	0.005	0.41 ^b^	0.05
	M	0.56 ^b^	0.04	0.07 ^ab^	0.006	0.59 ^a^	0.06
	N	0.63 ^b^	0.03	0.10 ^a^	0.003	0.32 ^b^	0.02
	O	0.70 ^a^	0.05	0.09 ^ab^	0.01	0.79 ^a^	0.07
	P	0.56 ^b^	0.02	0.05 ^c^	0.006	0.41 ^b^	0.03
*A. arbuscula*	Treatment	N (%)	SE	P (%)	SE	K (%)	SE
	I	0.60 ^b^	0.02	0.06 ^b^	0.003	0.55 ^b^	0.03
	J	0.77 ^a^	0.02	0.06 ^b^	0.006	0.43 ^b^	0.03
	K	0.65 ^b^	0.00	0.06 ^b^	0.003	0.33 ^b^	0.01
	L	0.62 ^b^	0.02	0.06 ^b^	0.004	0.41 ^b^	0.03
	M	0.55 ^b^	0.03	0.06 ^b^	0.003	0.34 ^b^	0.01
	N	0.77 ^a^	0.01	0.10 ^a^	0.003	0.32 ^b^	0.01
	O	0.71 ^b^	0.07	0.08 ^a^	0.01	0.69 ^a^	0.12
	P	0.54 ^b^	0.01	0.05 ^b^	0.004	0.41 ^b^	0.06
*A. nova*	Treatment	N (%)	SE	P (%)	SE	K (%)	SE
	I	0.59 ^b^	0.04	0.07 ^a^	0.01	0.40 ^b^	0.06
	J	0.59 ^b^	0.03	0.09 ^a^	0.008	0.35 ^b^	0.02
	K	0.57 ^b^	0.04	0.09 ^a^	0.007	0.43 ^b^	0.08
	L	0.58 ^b^	0.05	0.06 ^a^	0.01	0.40 ^b^	0.01
	M	0.82 ^a^	0.01	0.07 ^a^	0.003	0.73 ^a^	0.07
	N	0.70 ^a^	0.04	0.08 ^a^	0.003	0.26 ^b^	0.02
	O	0.69 ^b^	0.04	0.08 ^a^	0.01	0.51 ^ab^	0.07
	P	0.5 ^b^	0.04	0.06 ^a^	0.01	0.43 ^b^	0.04

## Data Availability

Not applicable.
